# Associations of Anemia With Outcomes in Patients With Spontaneous Intracerebral Hemorrhage: A Meta-Analysis

**DOI:** 10.3389/fneur.2019.00406

**Published:** 2019-04-25

**Authors:** Shuting Zhang, Xiaohua Pan, Chenchen Wei, Lu Wang, Yajun Cheng, Zhi Hu, Wei Dong, Ming Liu, Bo Wu

**Affiliations:** ^1^Department of Neurology, West China Hospital of Sichuan University, Chengdu, China; ^2^Department of Neurology, Baotou Central Hospital, Baotou, China; ^3^Department of Neurological Intensive Care Unit, West China Hospital of Sichuan University, Chengdu, China

**Keywords:** meta-analysis, intracerebral hemorrhage, anemia, hemoglobin, mortality, poor outcomes

## Abstract

**Background:** Intracerebral hemorrhage (ICH) is a highly lethal disease without effective therapeutic interventions. Anemia is prevalent in neurocritical disease and correlated with higher mortality in the intensive care unit. However, there is a lack of evidence concerning the association between anemia and the clinical outcomes of ICH.

**Object:** We aimed to assess the association between anemia and outcomes in patients with ICH.

**Methods:** We systematically searched the Cochrane Library, MEDLINE, EMBASE and Web of Science from inception to November 2017. Eligible studies were cohort studies exploring the association between anemia and mortality or functional outcomes in patient with ICH. A Meta-analysis was performed, and heterogeneity was assessed using the I^2^ index. Sensitivity analyses were performed to account for heterogeneity and risk of bias. Effect estimates were combined using random effects model for mortality and poor outcomes.

**Results:** We identified seven cohort studies with 7,328 ICH patients, including 1,546 patients with anemia. The meta-analysis revealed that anemia was associated with higher mortality {OR = 1.72 for 30-day mortality (95% CI 1.37 to 2.15; I^2^ = 64%; low-quality evidence); OR = 2.05 for 12-month mortality (95% CI 1.42 to 2.97; I^2^ = 82%; low-quality evidence)} and an increased risk of poor outcome in patients with ICH {OR = 2.29 for 3-month outcome (95% CI 1.16 to 4.51; I^2^ = 91%; very low-quality evidence); OR = 3.42 for 12-month outcome (95% CI 0.50 to 23.23; I^2^ = 96%; very low-quality evidence)}.

**Conclusions:** Anemia on admission was associated with higher mortality and an increased risk of poor outcome in patients with ICH. However, the results were limited by the high heterogeneity of included studies. Prospective, multi-center or population-based, large sample cohort studies are needed in the future.

## Introduction

Intracerebral hemorrhage (ICH) is the second most common cause of stroke and a highly lethal disease (1), which still lacks effective therapeutic interventions (2, 3). Although age, baseline ICH volume and neurological status on admission are well-known predictors of outcome of ICH (4), none of these factors could be easily manipulated in clinical practice to improve the outcome. Hemoglobin level, cerebral blood flow and arterial oxygen saturation determine the arterial oxygen content, which is essential for oxygen supply and autoregulation of small arteries in essential organs such as the brain and heart (5). Anemia is prevalent in the intensive care unit and is frequently associated with poor outcome in neurological disease such as ischemic stroke, aneurysmal subarachnoid hemorrhage (aSAH) and ICH (6, 7). It was plausible that low hemoglobin level and anemia might adversely affect the outcome of patients with ICH.

Although anemia has been correlated with poorer outcome in patients with ischemic stroke and subarachnoid hemorrhage(6, 8–10), there was a lack of evidence concerning the correlation between anemia and outcomes in patients with ICH. It was reported that anemia was correlated with poorer outcome in patients with ICH (8–10), however, these studies were conducted in different designs with inconsistent conclusions. To determine whether an association exists between anemia and outcomes in patients with ICH, we conducted a systematic review of the literature and performed non-randomized meta-analysis.

## Methods

### Search Strategy

Two reviewers (STZ and CCW) independently performed a literature research through the Cochrane Library, MEDLINE, EMBASE, and Web of Science from inception to November 2017. The following searching terms were used: “intracerebral hemorrhage,” “stroke,” “anemia,” “hemoglobin”. The search was limited to the English language.

### Study Selection

Studies of anemia and outcomes in patients with non-traumatic ICH that included patients of any age and either sex were eligible. ICH was defined as spontaneous intra- parenchymal bleeding with or without intraventricular extension. Patients were excluded as (1) non-strokes if they were diagnosed with traumatic ICH, primary subdural/epidural hematoma, intracranial venous thrombosis, or hemorrhage due to a tumor or recurrent ICH; (2) stroke due to primary subarachnoid hemorrhage with or without ICH and hemorrhagic transformation of a cerebral infarction. Anemia was defined as a hemoglobin concentration below 120 g/L (7.5 mmol/L) in females and below 130 g/L (8.1 mmol/L) in males following the WHO criteria (4). Outcomes were compared between anemia and non-anemia groups. The studies had to report the outcomes as either mortality or functional outcomes at a specified time point (e.g., 3 months or 12 months). The functional outcome was assessed using a modified Rankin scale (mRS).

### Study Assessment and Data Extraction

Two reviewers (STZ and CCW) independently performed the study assessment and data was reported in accordance with the Preferred Reporting Items for Systematic reviews and Meta-Analyses (PRISMA) statement (11). Study quality was assessed using the modified version of the Newcastle-Ottawa Scale (NOS) (12). A score of up to 9 points was assigned to each study based on the quality of anemia and non-anemia group selection, comparability between groups and assessment of outcome, such as mortality and mRS score. Baseline features were extracted from each study in Supplementary Table 2. ICH was confirmed by CT and ICH volume was determined by the formula for ellipsoids (A^*^B^*^C/2) (13). Mortality was based on mRS score of 6. For mortality within 30 days, only in-hospital mortality was reported in one study and was treated as 30-days mortality in the meta-analysis (10). Poor outcome was defined as an mRS score of 3 to 6 and favorable outcome was defined as an mRS score of 0 to 2. The primary outcomes were mortality and poor functional outcome. The raw numbers were extracted from each outcome of patients with and without anemia. In studies where adjusted ORs were obtained using multivariable analysis, the covariates were collected.

### Data Analysis

We calculated crude odds ratio (OR) and 95% confidence interval (CI) for dichotomous data and weighted mean difference (WMD) for continuous variables. Random-effect model was used to presume that the true effect size varied among studies and fixed-effect model was also applied in the sensitivity analysis to test the stability of pooled results. Heterogeneity among studies was assessed using the I^2^ index. According to the Cochrane handbook, the heterogeneity was classified as low (I^2^ index being 0–40%); moderate (30–60%); substantial (50–90%); considerable (75–100%) (14). When the outcome included more than 3 studies, we conducted sensitivity analysis using the “leave-one-out” approach to test the contribution of individual study to the pooled results. Egger tests and Funnel plots were used to evaluate publication bias (15). We then performed trim and fill analysis to recalculate the summary effect size if *p*-value of Egger tests were < 0.05 (16). Two reviewer (STZ and LW) independently applied Grading of Recommended Assessment, Development and Evaluation (GRADE) system to evaluate the quality of evidence (17). Each outcome was classified as high-quality, moderate-quality, low-quality, or very low-quality on the basis of GRADE system. All calculations were carried out using statistical software provided by the Cochrane Collaboration (RevMan 5.1) and Stata14.1 (StataCorp. 2015. Stata Statistical Software: Release 14. College Station, TX: StataCorp LP).

## Results

### Patient Characteristics

**Figure 1:**
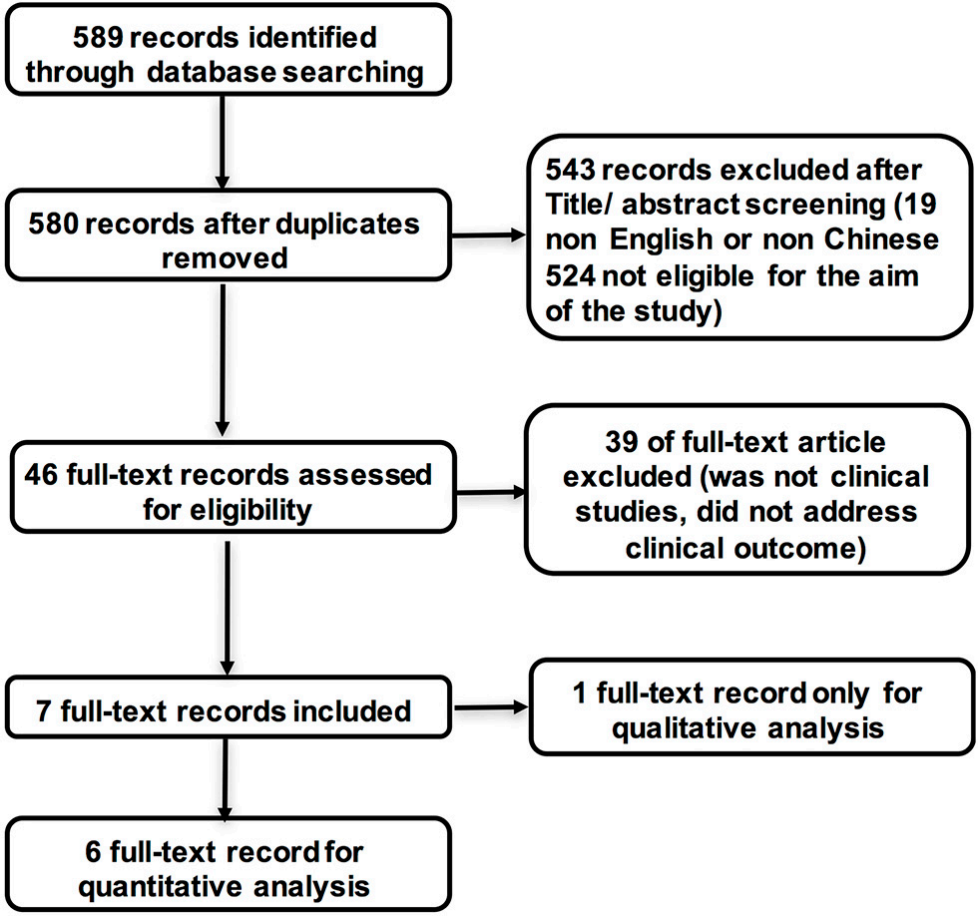
Flow chart of the study selection process.

The search strategy identified 580 studies without duplications and 543 studies were excluded based on abstract screening. Of 46 studies with full text, 39 studies were excluded for not being clinical studies or for lack of description of clinical outcome. We finally included 7 studies with 7,328 patients with ICH in this meta-analysis (Figure 1). The study quality was assessed using the modified NOS criteria. Except for one study that received a quality score of 7 out of 9, all studies received scores between 8 and 9 (Supplementary Table 1).

Basic characteristics of the seven studies are summarized in Table 1. Except Diedler (8), six observational cohort studies, comprising 1,546 patients with anemia and 5,586 patients without anemia, were included into the quantitative meta-analysis (9, 10, 18–21). Only one study recruited participants from a multicenter stroke registration (20). All participants were in-hospital patients with ICH except one study (21) including both ischemic stroke and ICH patients in two separate cohorts. The mean age of participants ranged from 59.8 to 77.8 years. The percentage of females was 33.7 to 52.4%. Follow-up periods varied among the included studies, ranging from 1 month to 12 months. Except in two studies reporting mortality only (9, 21), clinical outcomes were measured by both functional impairment (e.g., mRS) and mortality rates in other studies.

**Table 1:** Baseline characteristics of included studies.

**References**	**Start**	**Design**	**ICH Type**	**No. of Subjects**	**Mean age**	**Female**	**Prevalence of anemia**	**Mean of hemoglobin**	**ICH volume**	**Outcome definition**	**NIHSS score**	**Follow-up (Losses to follow-up, %)**
Kumar et al. (9)	1999–2005	Pro.	Supra.	685	71.3	44.8%	25.84%	135.8 g/L	**Anemia:** 42.7 ml (IQR 1.0–167.0)	30-days mortality	NOA	1 month (1%)
									**Non-anemia:** 34.3 ml (IQR 1.0–159.0)			
Diedler et al. (8)	2004–2004	Retro.	Supra.	196	67.1	33.7%	NOA	137 g/L	**mRS 4-6:** 13.6 ml (IQR 66.4)	mRS4-6	NOA	3 month (10%)
									**mRS 2-3:** 8.6 ml (IQR 24.0)			
Kuramatsu et al. (10)	2006–2010	Pro.	Supra.& Infra.	435	69.7	45%	24.14%	130 g/L	**Anemia:** 48.6 ml (IQR 12.1-82.2) **Non-anemia:** 15.0 ml (IQR 4.8-40.6)	mRS	Anemia: 22(15–32)Non-anemia: 14(5–24)	12 month (0%)
Bussiere et al. (18)	2003–2008	Pro.	Supra.& Infra.	2389	68.0	46.3%	22.69%	138.0g/L	NOA	mRS4-6 and mortality	CNS Score	12 month (0%)
Chang et al. (19)	2008–2010	Retro.	Supra.& Infra.	109	59.8	45.9%	27.53%	110.9g/L	NOA	mRS	Anemia: 16(0–40)	NOA
											Non-anemia: 14(1–40)	
Zeng et al. (20)	2007–2008	Pro.	Supra.& Infra.	2513	61.9	38.7%	19.30%	NOA	Anemia^*^: 14.71 ml (IQR 6.24–30.24)	mRS4-6 and mortality	Anemia: 10 (4–17)	12 month (8%)
											Non-anemia: 9 (3–16)	
									Non-anemia^*^: 14.85 ml (IQR 6–29.4)			
Barlas et al. (21)	2003–2015	Pro.	Supra.& Infra.	1001	77.8	52.4%	21.58%	NOA	NOA	mortality	NOA	12 month (?%)

*Supra, supratentorial; Infra., infratentorial; Pro., prospective; Retro., retrospective*;

* *, specifically for supratentorial ICH volume; mRS, modified Rankin Scale*.

### Mortality

**Figure 2:**
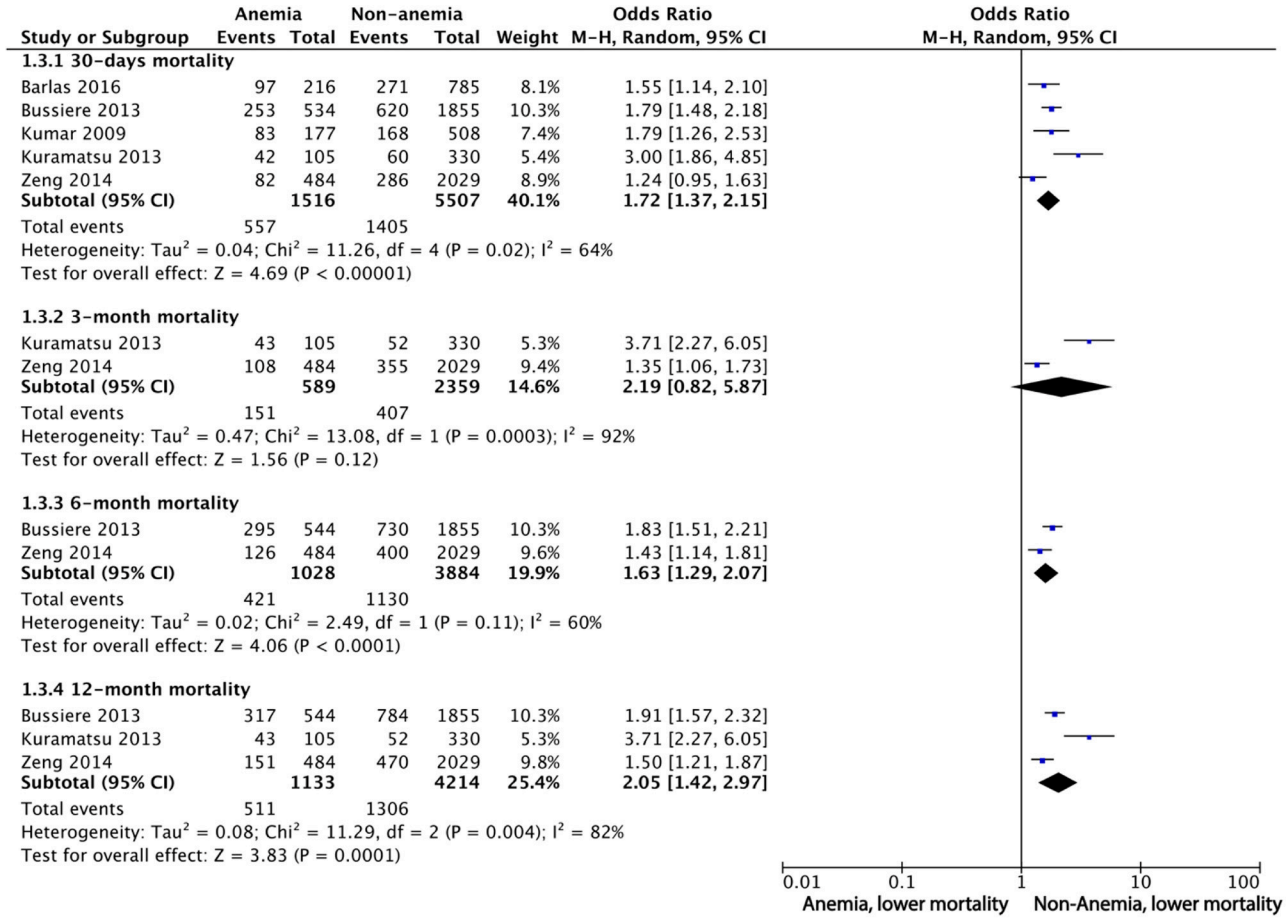
Meta-analysis of dichotomous data on the relationship between anemia and mortality using random-effect model.

Seven studies reported mortality at different time points. Five of them were included in the qualitative meta-analysis, because the other two studies reported the adjusted OR of mean hemoglobin or nadir hemoglobin instead of anemia. Adjusted ORs for mortality were obtained from four studies (Table 2). For 30-day mortality, the pooled OR of anemia was 1.72 (95%CI 1.37 to 2.15), with substantial heterogeneity (I^2^ = 64%, 5 studies, 7,023 participants, low-quality evidence). For 3-month mortality, the pooled OR was 2.19 (95%CI 0.82 to 5.87), with considerable heterogeneity (I^2^ = 92%, 2 cohorts, 2,948 participants, very low-quality evidence). For 6-month mortality, the pooled OR for mortality was 1.63 (95%CI 1.29 to 2.07) with substantial heterogeneity (I^2^ = 60%) (2 cohorts with 4,912 participants, very low-quality evidence). For 12-month mortality, the pooled OR was 2.05 (95%CI 1.42 to 2.97) with considerable heterogeneity (I^2^ = 82%, 3 cohorts with 5,347 participants, low-quality evidence) (Figure 2).

**Table 2:** Association between anemia or hemoglobin and clinical outcome of the studies included in the systematic analysis.

**References**	**Outcome definition**	**Follow-up** **(Losses to follow-up, %)**	**No. of Subjects**	**Unadjusted OR for poor outcome (CI)**	**Adjusted oR (CI)**	**Adjusted factors**
Kumar et al. (9)	30-days mortality	1 month (1%)	685	30-days mortality: 1.7 (1.2–2.4)	30-days mortality: 1.5 (0.9–2.4)	1,2,3,4
Diedler et al. (8)	mRS 4-6	3 month (10%)	196	NOA	Poor outcome: 0.73 (0.58–0.92)*	1,3,4,5,6,7
Kuramatsu et al. (10)	mRS 4-6 and mortality	12 month (0%)	435	1-year mortality:2.4 (1.12–5.1)	Poor outcome: 3.05 (1.31–7.06)	1,3,4
				1-year poor outcome: 7.5 (3.87–14.48)		
Bussiere et al. (18)	mRS 4-6 and mortality	12 month (0%)	2389	NOA	**1-year mortality:** 0–100 vs. 141–160: 1.39 (1.01–1.91)	1,2,4,5
					101–120 vs. 141–160: 1.15 (0.94–1.41)	
Chang et al. (19)	mRS	NOA	109	NOA	In-hospital mortality: 1.12 (0.917–1.364)	1,6,7
Zeng et al. (20)	mRS4-6 and mortality	12 month (8%)	2513	30-days mortality: 1.243 (.951–1.626)	3-months poor outcome: 1.04 (0.80–1.36)	1,2,3,5,6,8
				3-months mortality: 1.355 (1.063–1.727)	6-months poor outcome: 1.20 (0.92–1.57)	
				6-months mortality: 1.434 (1.138–1.805)	1-year poor outcome: 1.11 (0.85–1.43)	
				1-year mortality: 1.504 (1.209–1.871)	30-days mortality: 1.21 (0.87–1.68)	
				3-months poor outcome: 1.259 (1.032–1.536)	3-months mortality: 1.20 (0.89–1.62)	
				6-months poor outcome: 1.39 (1.139–1.696)	6-months mortality: 1.34 (1.01–1.78)	
				1-year poor outcome: 1.349 (1.106-1.646)	1-year mortality: 1.33 (1.00-1.75)	
Barlas et al. (21)	mortality	12 month (NOA)	1001	NOA	**Male:** 1-month mortality: 1.42 (0.83–2.42)	1,5,8
					3-month mortality:1.39 (0.81–2.39)	
					6-month mortality:1.64 (0.94–2.85)	
					12-month mortality:1.76 (1.01–3.04)	
					**Female:** 1-month mortality: 1.29 (1.04–1.60)	
					3-month mortality: 1.39 (1.14–1.70)	
					6-month mortality: 1.44 (1.18–1.75)	
					12-month mortality: 1.48 (1.23–1.79)	

mRS, modified Rankin Scale; NOA, not available; *, mean hemoglobin was compared between groups; NIHSS, National Institute of Health stroke scale. We record “1” for study which adjusted for age, “2” adjusted for sex, “3” adjusted for ICH volume, “4” adjusted for IVH, “5” adjusted for drug use, “6” adjusted for NIHSS, “7” adjusted for transfusion, “8” adjusted for other vascular risk factors including coronary heart disease, congestive heart failure, atrial fibrillation, hypertension, hyperlipidemia, diabetes mellitus.

**Figure 3:**
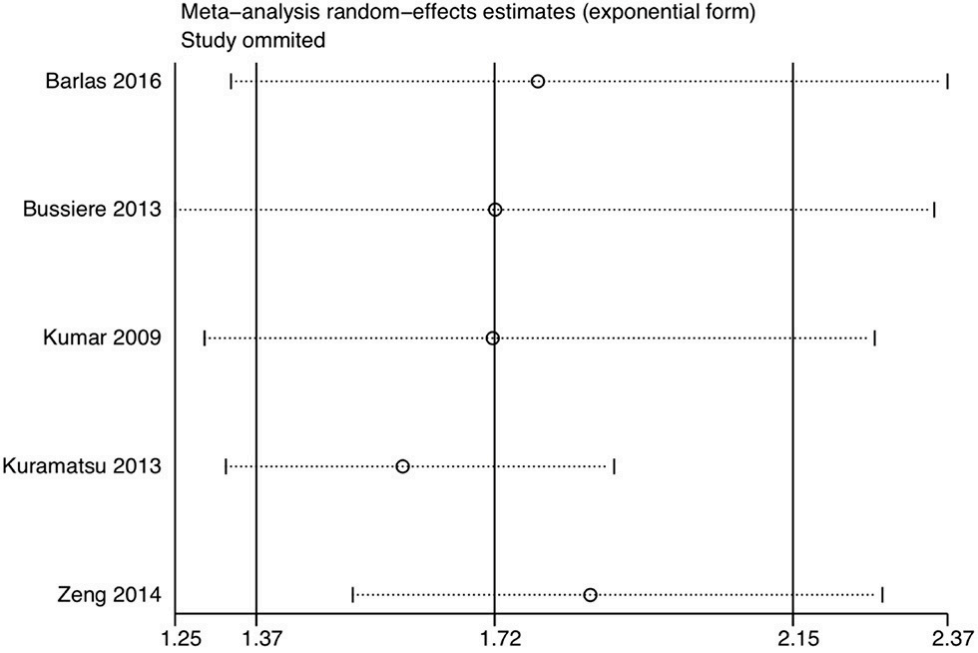
Sensitivity analysis of 30-days mortality using the leave-one-out method.

We also performed sensitivity analysis by combining the pooled ORs of fixed-effect model, which were similar to the results of random-effect model except for 3-month mortality with OR being 1.64 (95%CI 1.33 to 2.03) (Supplementary Figure 1). Sensitivity analysis of “leave-one-out” approach indicated none of the results of the single study were obviously different from others after removing studies one by one (Figure 3 and Supplementary Figure 3). There was no significant evidence of publication bias for all the outcomes (Supplementary Figure 5: Funnel plot of 30-days mortality). The detailed evidence quality assessment of GRADE system was displayed in Supplementary Table 2.

### Poor Outcome

Five studies reported poor outcomes with different follow-up periods. The definition of poor outcome varied. For the meta-analysis, we defined poor outcome as an mRS of 3 to 6 and collected corresponding data according to the published data. Two studies compared the hemoglobin level as a continuous variable among different mRS grades, thus eliminating themselves from the qualitative meta-analysis(8, 19). Diedler et al. demonstrated an adjusted OR of 0.73 (95%CI 0.58 to 0.92) for 3-month poor outcomes (an mRS score of 4–6) when mean hemoglobin was treated as a continuous variable, indicating the lower the hemoglobin, the poorer the outcome. Chang et al. demonstrated an adjusted OR of 1.429 (95%CI 1.055 to 1.938) for discharge poor outcome defined as an mRS score of 5–6 when nadir hemoglobin during hospitalization was compared between poor and other outcome groups. The adjusted OR for poor outcome was obtained from five studies (Table 2). Overall, all five studies suggested a significant impact of anemia on poor outcomes of ICH regardless of the definition of the poor outcomes and the grouping methods.

**Figure 4:**
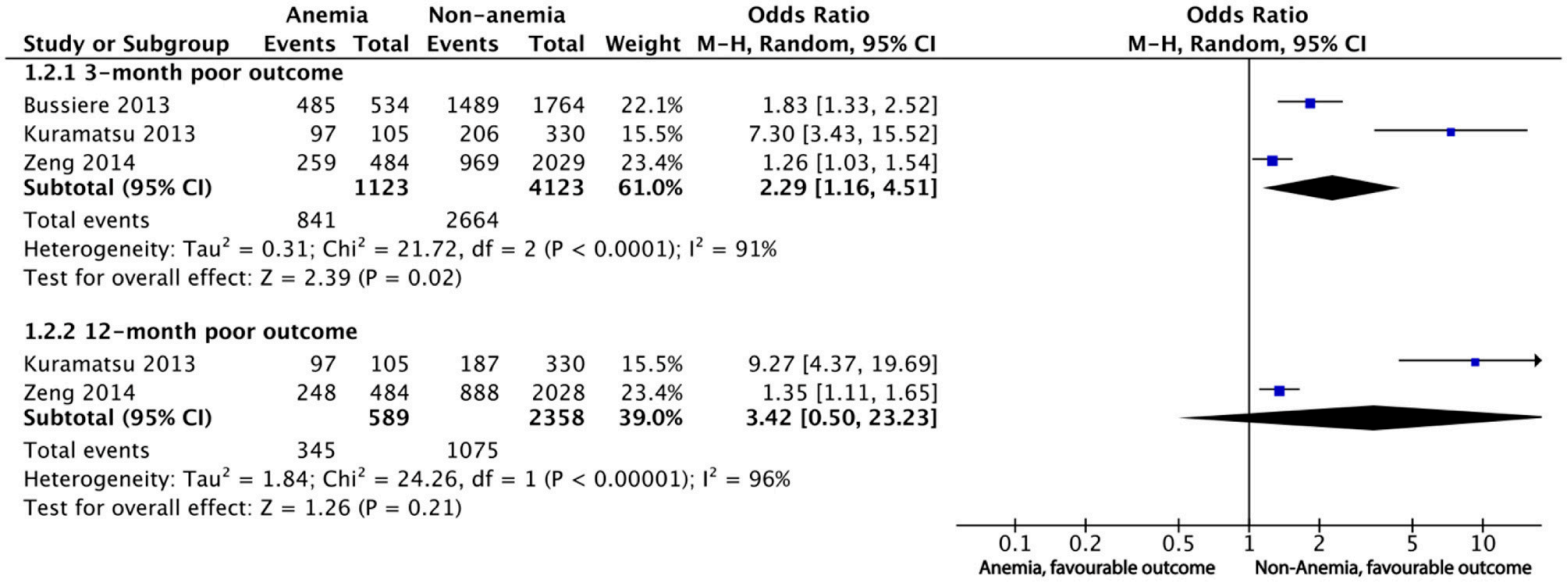
Meta-analysis of dichotomous data on the relationship between anemia and poor outcome.

For short-term poor outcomes, including the outcomes within 3 months, the pooled OR for poor outcome was 2.29 (95%CI 1.16 to 4.51), with considerable heterogeneity (I^2^ = 91%, 3 cohorts, 5,246 participants, very low-quality evidence). The pooled ORs for long-term poor outcome was 3.42 (95%CI 0.50 to 23.23) with considerable heterogeneity (I^2^ = 96%) when 12-month poor outcome was compared between anemia and non-anemia groups (2 cohorts with 2,947 participants, very low-quality evidence) (Figure 4 and Supplementary Table 2). When we estimated the effect size using fixed-effect model, the result of 3-month poor outcome was still robust but the result of 12-month poor outcome changed significantly {OR and 95%CI being 1.66 (95%CI 1.38 to 2.00)} (Supplementary Figure 2). When testing the contribution of individual study to the main pooled outcomes, no study was significantly different from other studies (Supplementary Figure 4). However, the asymmetry funnel plot revealed the existence of potential publication bias for the 3-month poor outcome (*P* = 0.009), while no missing study added in the trim and fill (Supplementary Figure 6).

## Discussion

This systematic review and meta-analysis demonstrated that in patients with ICH, anemia was significantly associated with higher mortality and an increased risk of poor functional outcome compared with non-anemia, but considerable heterogeneity was observed. According to GRADE system, the SOE (strength of evidence) for the associations of anemia and clinical outcomes were low or insufficient. Therefore, the results should be interpreted with caution. On this point, we suggested that the causality of anemia and the increased risk of poor outcome cannot be established in patients with ICH. High-quality, multi-center, large sample cohort studies are needed to address the questions in the future.

The findings from this study was consistent with a recent meta-analysis, indicating a positive correlation between anemia and higher mortality in stroke patients {pooled OR 1.39 (95%CI 1.22–1.58)} (22). However, the previous meta-analysis included patients with ischemic stroke and ICH. The patients with ICH distinguish from ischemic stroke patients in various aspects, such as sex, comorbidities and the underlying pathologic mechanisms (23). Our present study focused on the patients with ICH and provided information regarding anemia and outcomes in ICH, and included recently published studies (21). Although the mechanisms underlying the association between anemia and poorer outcome in ICH was still unknown, several studies suggested the correlation of anemia and abnormal coagulation, hemostatic alterations, and an increased bleeding tendency (24, 25). It is possible that the disturbed hemostasis in anemia was associated with abnormal vascular modulation since erythrocyte and hemoglobin could act as vascular modulator via activating endothelium-derived vasoconstriction (26). Furthermore, anemia was frequently correlated with iron deficiency, which was correlated with microcytosis and iron-deficiency-activated hypercoagulability (24, 25). Other detrimental anemia-based mechanisms refer to neuronal tissue hypoxia, metabolic distress, and cell energy dysfunction that possibly lead to pronounced secondary cerebral injury by a reduced oxygen carrying capacity (27–29).

The strengths of this study should be highlighted. First, we applied a comprehensive search strategy without language restriction to ensure that the included studies were representative. Second, our study adheres to the standard methodology of systematic review and meta-analysis as required by the Cochrane and PRISMA checklist (11). Third, both random and fixed effects models were performed to estimate the effect size of the association between anemia and outcomes at different time points. Fourth, we graded the SOE for the association between anemia and outcomes in ICH using the GRADE system and further estimate the strength of the conclusion in this meta-analysis. Fifth, the sample of all included studies is moderate and the quality of them is good according to the NOS criteria. Finally, we conducted sensitivity analyses using the leave-one-out method. Generally, no study significantly changes the conclusions in 30-days mortality, 12-month mortality and 12-month poor functional outcome. Except 3-month poor outcome, funnel plot and Egger's test revealed no significant publication bias in the analysis of other outcomes. These analyses suggested that, based on the existing studies, the results of this meta-analysis was relatively stable.

Our study also has limitations. Firstly, all included studies in the meta-analysis were observational studies, which are prone to bias and unmeasured confounders. There might be indication bias in the anemia group. For instance, patients with anemia tend to be more severe, and are more likely to be transferred to ICU and affected by the limitation of care orders (LCOs). The LCOs could exacerbate already poor outcome in anemia patients, which leads to confounding by indication and overestimates the detrimental effects of anemia on ICU (30). Although at least two studies included patients in ICU, none of them mentioned the effect of LCOs on the outcomes of ICH. Secondly, anemia might represent a surrogate for comorbidities. Three included studies adjusted comorbidity variables in the multivariable analysis, but different comorbidities were considered in each study (Table 2). However, our meta-analysis of comorbidities demonstrated that, except diabetes, there was no significant difference of comorbidities between anemia and non-anemia groups (Table 2 and Supplementary Figure 7). Moreover, no study reported the comorbidities associated with the occurrence of anemia, such as coagulation dysfunction, chronic renal failure and abnormal liver function, especially. Thirdly, given the significantly lower hemoglobin level in females, it is plausible that the correlation of anemia and outcomes of ICH is different by gender. Future studies should consider the hemoglobin-associated factors like gender, comorbidities and LCOs when designing the protocol and analyzing the data. Finally, the high heterogeneity in this meta-analysis may limit our findings. Although sensitivity analysis revealed no single study significantly influenced the effect size of the outcomes, Zeng (20) distinguish itself in several aspects. This study was characterized with relatively negative results with small OR. One possibility might be it is the only study of Asian population, and the difference between Asian and Caucasian population might influence the association between anemia and outcomes in ICH. These variable results indicate that a well-designed, large sample sized cohort study is needed to help confirm the associations presented in this review.

Although the meta-analysis demonstrated that anemia might increase the risk of poor outcome of ICH, few studies investigated the effect of packed red blood cell (PRBC) transfusion in ICH patients. In this review, only Diedler (8) and Chang (19) mentioned PRBC transfusion but not consider this factor in final analysis. In neurocritical diseases, PRBC transfusion was mostly studied in the context of subarachnoid hemorrhage (SAH) (31–34). We conduct a thorough literature search but found only one retrospective cohort study of 546 patients investigated whether PRBC transfusion was associated with better outcome in ICH (34), suggesting that PRBC transfusion was associated with improved survival at 30 days. The application of PRBC transfusion was limited for its risks outweighing the benefits (35, 36), despite that Zheng et al. recently conducted meta-analysis to assess the relationship of PRBC transfusion with in-hospital mortality in ICU patients and suggested that PRBC transfusion might not be associated with an increased risk of in-hospital death (37). Although hemoglobin concentrations as low as 70 g/L are well tolerated in the general ICU patients (38), animal studies and human physiologic and observational studies supported that such a severe degree of anemia could be harmful in the neurocritical care patients due to the high sensitivity of brain to oxygen. Kramer et al. suggested that the transfusion threshold could be 80 to 90 g/L in selected patients in the neurocritical care unit (39), however, the systematic review based on six studies of 537 neurocritical patients found that there was insufficient evidence to support the higher PRBC transfusion threshold in the neurocritical patients (40). In specific neurocritical disease like SAH, a multidisciplinary consensus panel of the Neurocritical Care Society strongly recommends that patients with SAH should receive PRBC transfusion to maintain a hemoglobin concentration >80 to 100 g/L (41, 42). At present, PRBC transfusion should be still cautiously applied in practice and multicenter randomized trials are needed to ended this controversy.

## Conclusion

Anemia on admission was associated with poorer outcome and higher mortality in patients with ICH. However, the results were limited by the high heterogeneity of included studies. More rigorous studies are needed to explore the impact of anemia on the outcomes of ICH.

## Author Contributions

XP, SZ, and CW searched the scientific literature and drafted the manuscript. ML and BW contributed to conception, design and data interpretation. LW, ZH, WD, and YC helped to collect the data and performed statistical analyses. ML and BW contributed to conception, design, data interpretation, manuscript revision for critical intellectual content and supervision of the study. All authors read and approved the manuscript.

### Conflict of Interest Statement

The authors declare that the research was conducted in the absence of any commercial or financial relationships that could be construed as a potential conflict of interest.
