# Supplementary material for: Associations of Anemia With Outcomes in Patients With Spontaneous Intracerebral Hemorrhage: A Meta-Analysis
Source: Front Neurol. 2019 Apr 25;10:406. doi: 10.3389/fneur.2019.00406 (PMC6494942; doi:10.3389/fneur.2019.00406)
Supplement: Supplementary file 1 [file Data_Sheet_1.docx]

| **Reference** | **Selection** | | | | **Comparability** | **Outcome** | | | **Total** |
| --- | --- | --- | --- | --- | --- | --- | --- | --- | --- |
|  | **Representativeness** | **Selection of controls** | **Exposure Ascertainment** | **Outcome before the study** |  | **Assessment** | **Length of follow-up** | **Follow-up**  **loss** |  |
| Kumar et al; 2009 | 1 | 1 | 1 | 1 | 2 | 1 | 1 | 1 | 9 |
| Diedler et al ; 2010 | 1 | 1 | 1 | 1 | 2 | 1 | 1 | 1 | 9 |
| Kuramatsu et al; 2013 | 1 | 1 | 1 | 1 | 2 | 1 | 1 | 1 | 9 |
| Bussiereet al; 2013 | 1 | 1 | 1 | 1 | 1 | 1 | 1 | 1 | 8 |
| Chang et al; 2013 | 1 | 1 | 1 | 1 | 1 | 1 | 1 | 0 | 7 |
| Zeng et al; 2014 | 1 | 1 | 1 | 1 | 2 | 1 | 1 | 1 | 9 |
| Barlas et al; 2016 | 1 | 1 | 1 | 1 | 2 | 1 | 1 | 1 | 9 |

Supplementary table 1: Quality of studies included (Newcastle-Ottawa quality assessment scale).


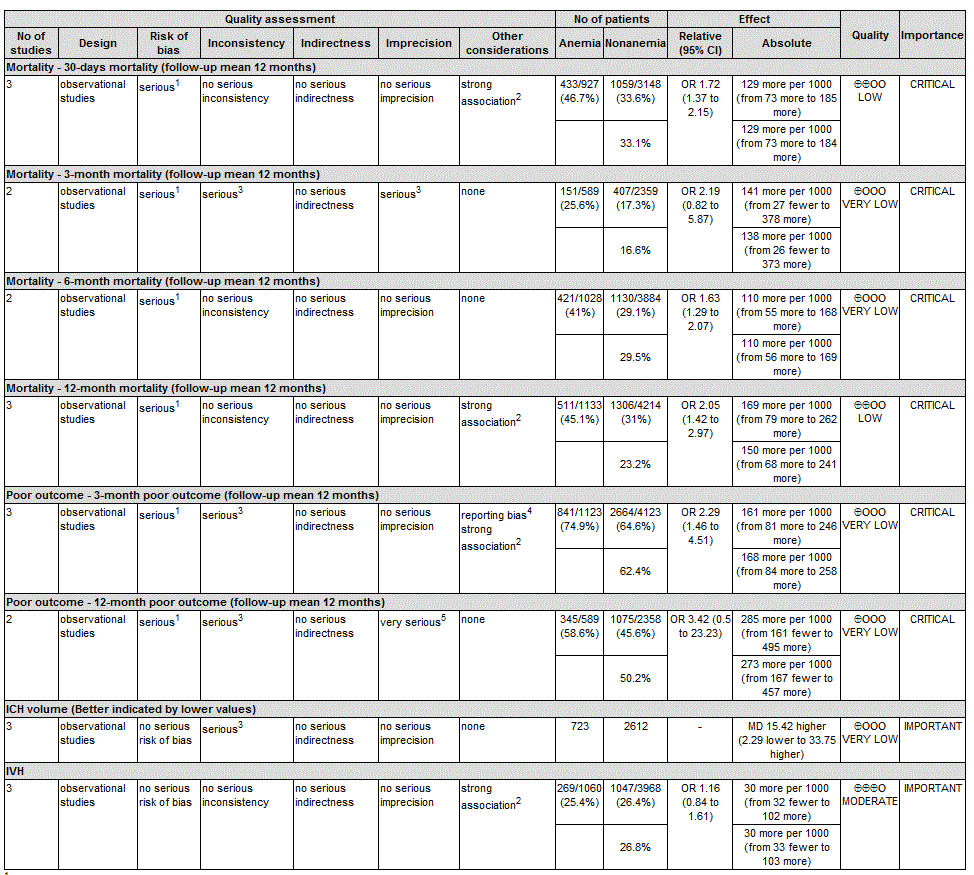
Supplementary table 2: GRADE summary of evidence for the effects of anemia and outcomes. 1, The study of Zeng 2014 had 8% of loss to follow-up rate; 2, Sample size was more than 5,000; 3, No explanation was provided; 4, The Egger test showed publication bias; 5, very wide confidence interval.


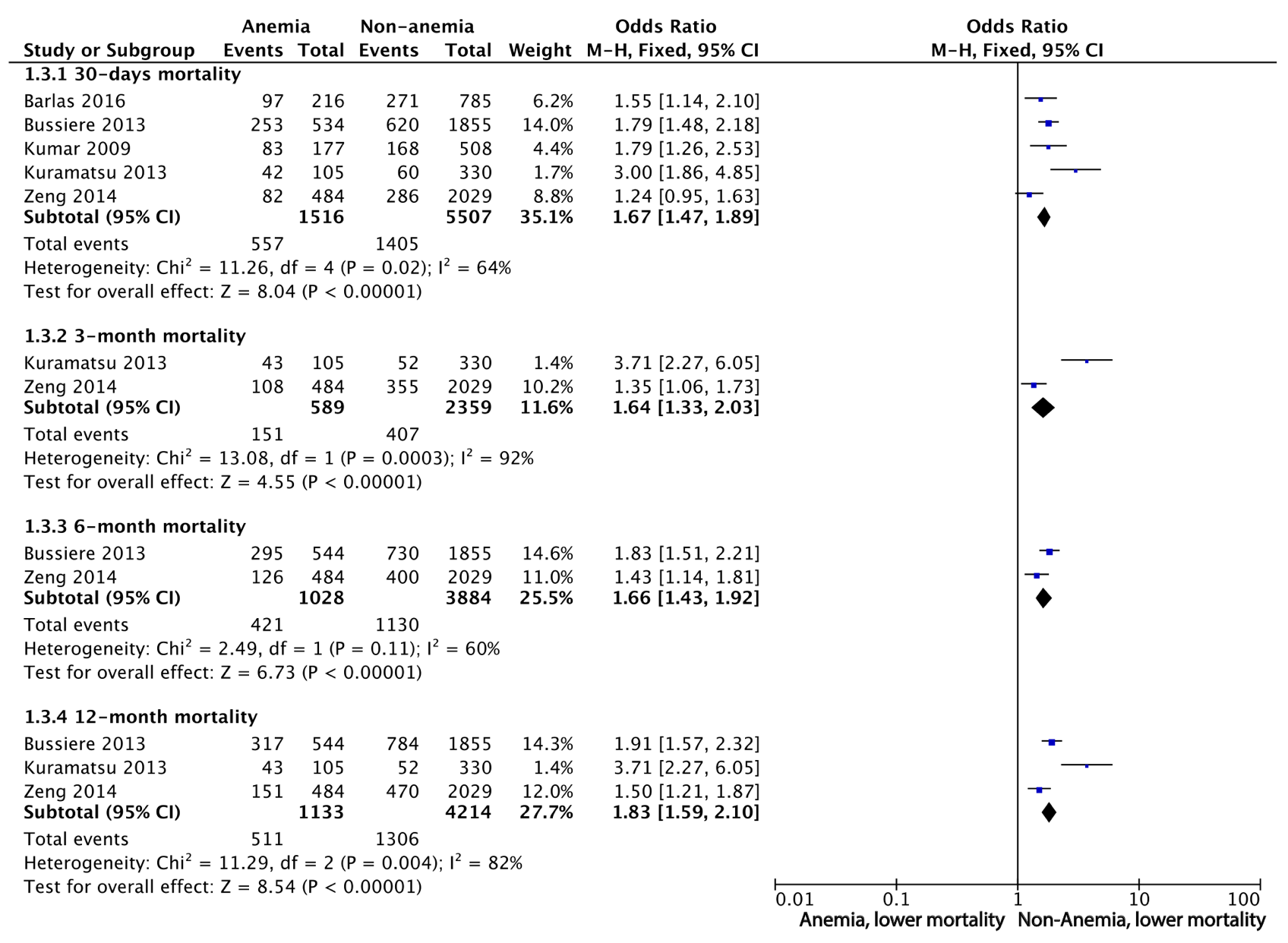
Supplementary figure 1: Sensitivity analysis by meta-analyzing the relationship between anemia and mortality using fixed-effect model.


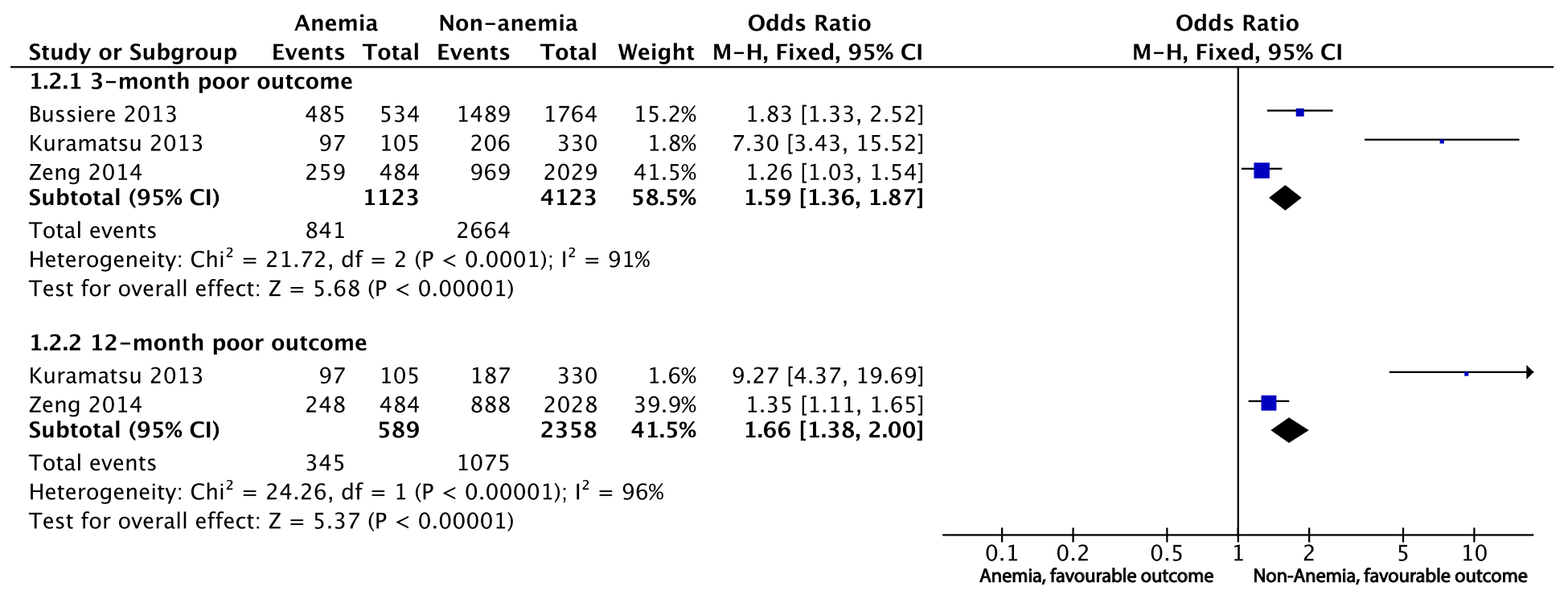


Supplementary figure 2: Sensitivity analysis by meta-analyzing the relationship between anemia and poor outcomes using fixed-effect model.


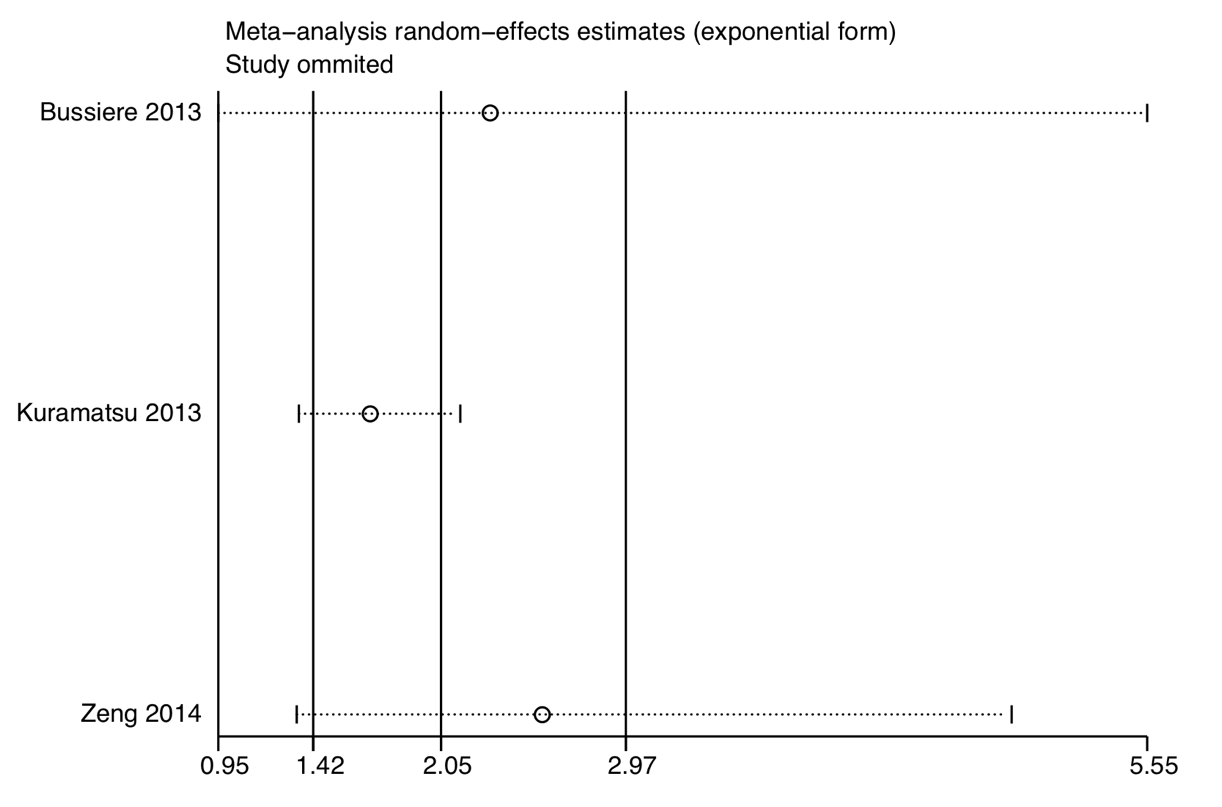


Supplementary figure 3: Sensitivity analysis of 12-month mortality using the leave-one-out method.

 Supplementary figure 4: Sensitivity analysis of 3-month poor outcome using the leave-one-out method.


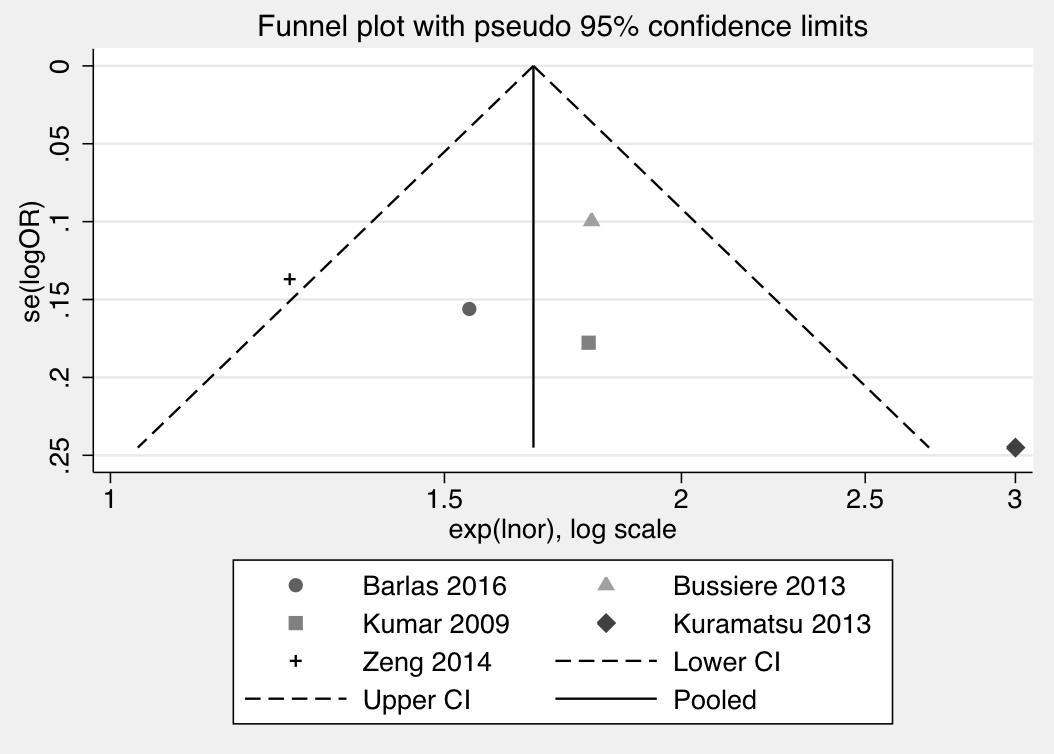


Supplementary figure 5: Funnel plot of 30-days mortality.


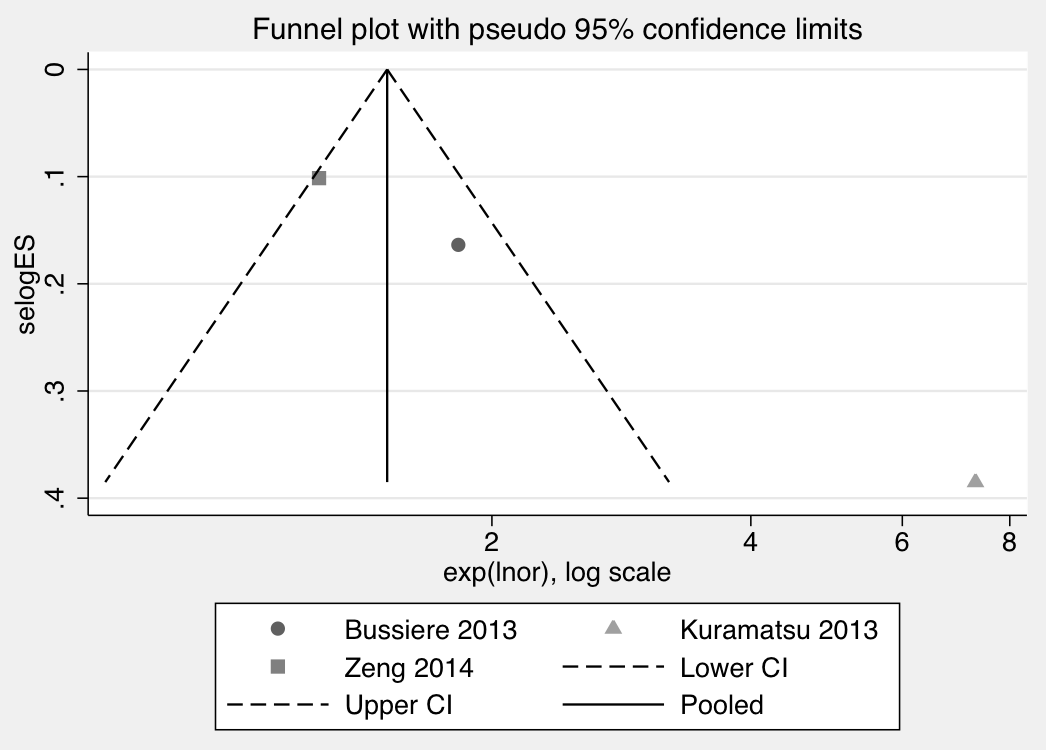


Supplementary figure 6: Funnel plot of 3-month poor outcome.


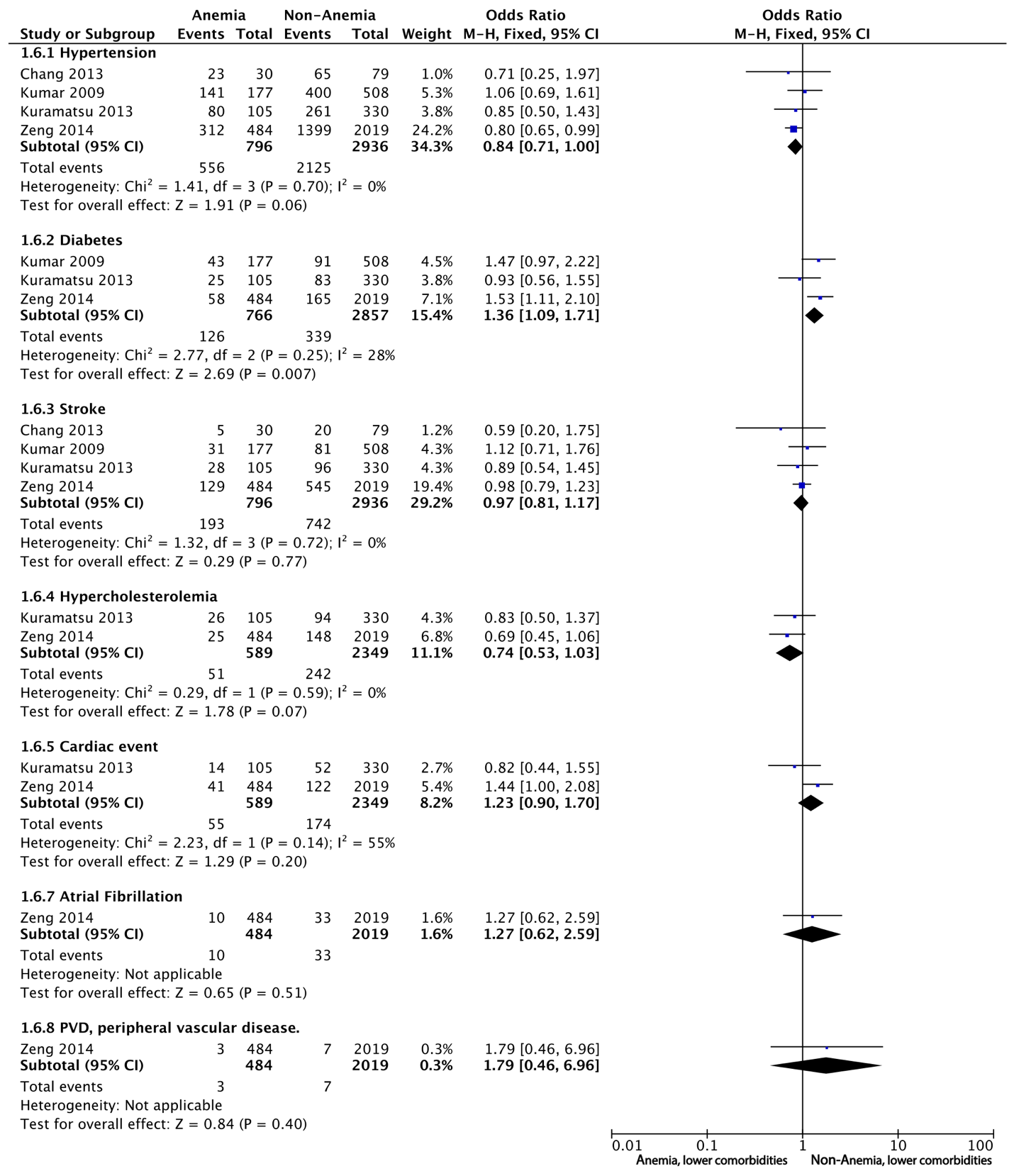
Supplementary figure 7: Meta-analysis of dichotomous data on the relationship between anemia and different comorbidities.
